# A novel kinetic model estimating the urea concentration in plasma during non-invasive sweat-based monitoring in hemodialysis

**DOI:** 10.3389/fphys.2025.1547117

**Published:** 2025-03-18

**Authors:** Xiaoyu Yin, Sophie Adelaars, Elisabetta Peri, Eduard Pelssers, Jaap Den Toonder, Arthur Bouwman, Daan Van de Kerkhof, Massimo Mischi

**Affiliations:** ^1^ Electrical Engineering, Eindhoven University of Technology, Eindhoven, Noord-Brabant, Netherlands; ^2^ Laboratory, Catharina Hospital, Eindhoven, Noord-Brabant, Netherlands; ^3^ Biomedical Engineering, Eindhoven University of Technology, Eindhoven, Noord-Brabant, Netherlands; ^4^ Anesthesiology, Catharina Hospital, Eindhoven, Noord-Brabant, Netherlands

**Keywords:** kidney failure, end-stage renal disease, patient monitoring, pharmacokinetic modeling, inverse modeling

## Abstract

**Introduction:**

The adequacy of hemodialysis (HD) in patients with end-stage renal disease is evaluated frequently by monitoring changes in blood urea concentrations multiple times between treatments. As monitoring of urea concentrations typically requires blood sampling, the development of sweat-sensing technology offers a possible less-invasive alternative to repeated venipuncture. Moreover, this innovative technology could enable personalized treatment in a home-based setting. However, the clinical interpretation of sweat monitoring is hampered by the limited literature on the correlation between urea concentrations in sweat and blood. This study introduces a pioneering approach to estimate blood urea concentrations using sweat urea concentration values as input.

**Methods:**

To simulate the complex transport mechanisms of urea from blood to sweat, a novel pharmacokinetic transport model is proposed. Such a transport model, together with a double-loop optimization strategy from our previous work, was employed for patient-specific estimation of blood urea concentration. 32 patient samples of paired sweat and blood urea concentrations, collected both before and after HD, were used to validate the model.

**Results:**

This resulted in an excellent Pearson correlation coefficient (0.98, 95%CI: 0.95–0.99) and a clinically irrelevant bias (−0.181 mmol/L before and −0.005 mmol/L after HD).

**Discussion:**

This model enabled the accurate estimation of blood urea concentrations from sweat measurements. By accurately estimating blood urea concentrations from sweat measurements, our model enables non-invasive and more frequent assessments of dialysis adequacy in ESRD patients. This approach could facilitate home-based and patient-friendly dialysis management, enhancing patient comfort while enabling more personalized treatment across diverse clinical settings.

## 1 Introduction

Monitoring urea concentrations in blood is essential for determining the adequacy of HD in patients with end-stage renal disease (ESRD). The necessity of frequent hospital visits for the HD treatment, often multiple times per week, significantly diminishes the quality of life of these patients. Home-based HD treatment could improve patients’ quality of life by reducing travel and providing a familiar environment. Additionally, customizing HD treatment to meet individual patient requirements could further optimize their treatment and, consequently, improve their wellbeing. To realize these personalized HD treatments in home settings, there is an urgent clinical need for developing non-invasive alternatives to frequent venipuncture. These alternatives should be capable of remote and continuous monitoring of biomarkers, both during and in intervals between HD sessions.

As a non-invasive biofluid, sweat contains a wide variety of biomarkers, including urea, presenting a promising alternative for assessing blood urea concentration. Although the measurement of urea concentrations in sweat is now achievable (Futane et al., 2023), the clinical consideration of sweat urea concentrations remains limited due to the unclear relationship between urea concentrations in sweat and blood. In previous work of our group and other recent literature, concentrations of urea in sweat are found to be somewhat higher than in plasma (Adelaars et al., 2024; Al-Tamer et al., 1997; Bulmer, 1957), implying that urea might not only diffuse passively over the different compartments. Possible explanations for the higher concentration in sweat include an additional urea source outside plasma like epidermal accumulation of urea (Brusilow, 1967; Gordon et al., 1976), the impact of evaporation on sweat urea concentrations, cleavage of arginine to urea in the sweat gland (Szondi et al., 2021; Baker et al., 2022), and active transport mechanisms via urea transporters in the sweat gland membrane (Xie et al., 2017). To our knowledge, no study has established a method to estimate plasma urea concentrations based on measurements in sweat. Advanced modeling of the urea transport mechanism from plasma to sweat would provide valuable insights into the kinetics of urea and facilitate the clinical interpretation of sweat urea monitoring results.

In a previous study of our group (Yin et al., 2024), we proposed a novel double-loop strategy using a glucose transport model that enables estimations of blood glucose concentrations based on sweat measurements in a personalized manner. It is important to note that this method is specifically designed for glucose monitoring, where the transport mechanism is known to be purely passive. Compared to glucose, urea transport kinetics between blood and sweat are more complex.

Building on the optimization framework introduced in our previous work (Yin et al., 2024), here we propose an innovative method that allows for the estimation of urea in blood using sweat urea concentrations, taking into account the complex kinetic behavior of urea. By incorporating physiological mechanisms into the modeling of urea transport, this approach offers a more robust clinical interpretation of sweat-based urea monitoring. This approach was tested on a population of ESRD patients with a large variation in urea concentrations across HD.

## 2 Materials and methods

### 2.1 Urea transport model

**FIGURE 1:**
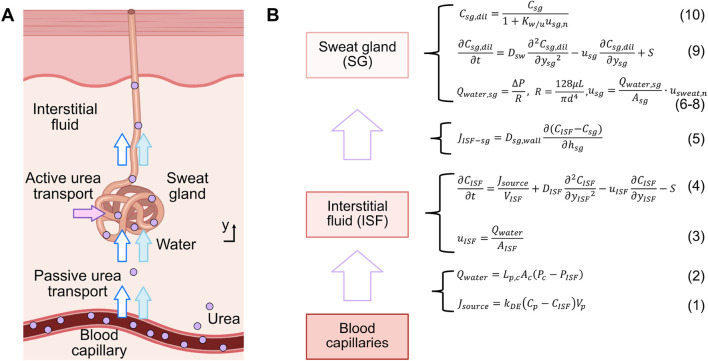
**(A)** Schematic illustration of the urea transport mechanism from blood to sweat along a single sweat gland, encompassing both passive and active transport. **(B)** Compartmental model and relevant formulas for urea transport.

To simulate the mechanism of urea transport from blood to sweat, a pharmacokinetic urea transport model was developed using COMSOL Multiphysics® software (Zoetermeer, Netherlands). This model builds upon the glucose transport model from previous work (Yin et al., 2024), which exclusively considered a passive transport mechanism based on convection and diffusion. The urea transport model is composed by three compartments: the blood capillary, interstitial fluid (ISF), and sweat gland. The overall transport process is summarized in Figure 1.

Firstly, a flow rate of urea from the blood capillary compartment to the ISF compartment, denoted as $J_{\mathrm{source}}$ in $mol\;s^{-1}$ , arises due to the concentration difference of urea between the plasma and the ISF. This process can be described as shown in Equation (1)

$$J_{\mathrm{source}}=k_{DE}\left(C_{p}-C_{\mathrm{ISF}}\right)V_{p},$$ (1)

where $C_{p}$ and $C_{\mathrm{ISF}}$ are the concentrations of urea in $mol\;m^{-3}$ in plasma and ISF, respectively, $k_{DE}$ is the dermal clearance constant of urea in $s^{-1}$ (Ibrahim et al., 2012), quantifing the rate at which urea is transferred from the capillary to the ISF, and $V_{p}$ is the effective volume of the blood capillary compartment related to a single sweat gland in $m^{3}$ (Himeno et al., 2016).

At the same time, due to the pressure difference between the blood capillary compartment and the ISF compartment, water flows from the blood capillary into the ISF at a flow rate denoted by $Q_{\mathrm{water}}$ in $m^{3}\;s^{-1}$ . This process is modeled by the Starling equation as shown in Equation (2)

$$Q_{\mathrm{water}}=L_{p,c}A_{c}\left(P_{c}-P_{\mathrm{ISF}}\right),$$ (2)

where $L_{p,c}$ represents the hydraulic conductivity in $m\;s^{-1}\;mmHg^{-1}$ (Kellen and Bassingthwaighte, 2003), $A_{c}$ represents the blood capillary compartment surface area in $m^{2}$ (Haggerty and Nirmalan, 2019), $P_{c}$ is the capillary hydrostatic pressure in mmHg (Haggerty and Nirmalan, 2019), and $P_{\mathrm{ISF}}$ is the interstitial hydrostatic pressure in mmHg (Haggerty and Nirmalan, 2019).

The water flow velocity in the ISF compartment, $u_{\mathrm{ISF}}$ (in $m\;s^{-1}$ ), is given by dividing the water flow rate, $Q_{\mathrm{water}}$ , by the cross-sectional area of the ISF compartment, $A_{\mathrm{ISF}}$ (in $m^{2}$ ), as shown in Equation (3)

$$u_{\mathrm{ISF}}=\frac{Q_{\mathrm{water}}}{A_{\mathrm{ISF}}}.$$ (3)

In the ISF compartment, urea undergoes a joint process of convection and diffusion, and a portion of it is also actively transported out this compartment. This active transport is facilitated by urea transporters located in the following sweat gland compartment (Xie et al., 2017). The transport process can be modeled as shown in Equation (4)

$$\frac{\partial C_{\mathrm{ISF}}}{\partial t}=\frac{J_{\mathrm{source}}}{V_{\mathrm{ISF}}}+D_{\mathrm{ISF}}\frac{\partial^{2}C_{\mathrm{ISF}}}{\partial y_{\mathrm{ISF}}^{2}}-u_{\mathrm{ISF}}\frac{\partial C_{\mathrm{ISF}}}{\partial y_{\mathrm{ISF}}}-S,$$ (4)

where $V_{\mathrm{ISF}}$ is the effective volume of the ISF compartment related to a single sweat gland in $m^{3}$ (Himeno et al., 2016), $D_{\mathrm{ISF}}$ is the urea diffusion coefficient in the ISF in $m^{2}\;s^{-1}$ (Steiner, 1981), and $y_{\mathrm{ISF}}$ is the distance between urea and the entrance of the ISF compartment in m . S represents the rate at which urea is actively transported out of the ISF compartment in $mol\;m^{-3}\;s^{-1}$ , serving as a sink term that reflects the active clearance rate of urea from the ISF compartment. This active transport removes urea from the ISF and transfers it into the sweat gland compartment, where the same term S acts as a source.

Thereafter, due to the concentration gradient between the ISF compartment and sweat gland compartment, urea is passively transported to the sweat gland compartment through diffusion across the gland wall. This process can be quantified by the urea flux $J_{\mathrm{ISF-sg}}$ in $mol\;s^{-1}$ , and can be described by the Fick’s first law as shown in Equation (5)

$$J_{\mathrm{ISF-sg}}=D_{sg,wall}\frac{\partial \left(C_{\mathrm{ISF}}-C_{sg}\right)}{\partial h_{sg}},$$ (5)

where $D_{sg,wall}$ in $m^{2}\;s^{-1}$ is the urea diffusion coefficient for the gland wall (Steiner, 1981), $C_{sg}$ is the concentration of urea in the sweat gland compartment in $mol\;m^{-3}$ , and $h_{sg}$ is the gland wall thickness in m (Sonner et al., 2015).

Water flows from the ISF compartment into the sweat gland compartment, and then to the skin’s surface, driven by the pressure difference ( ΔP in mmHg ) between the ISF and the external environment through the sweat gland (Schulz, 1969). The water flow rate $Q_{\mathrm{water,sg}}$ in $m^{3}\;s^{-1}$ , is described using Darcy’s law as shown in Equation (6)

$$Q_{\mathrm{water,sg}}=\frac{\Delta P}{R},$$ (6)

where R in $Pa\cdot s\;m^{-3}$ is the hydraulic resistance, and ΔP is the pressure difference under passive sweating conditions. The hydraulic resistance R is expressed as shown in Equation (7)

$$R=\frac{128\mu L}{\pi d^{4}},$$ (7)

where μ in Pa⋅s is water viscosity (Kestin et al., 1978), L in m is the length of the sweat gland compartment (Wilke et al., 2007), d in m is the luminal diameter of the sweat gland (Hibbs, 1958).

The pressure difference in Equations 2, 6 are closely related, with the interstitial pressure $P_{\mathrm{ISF}}$ acting as a common variable linking the water flow from the blood capillaries into the ISF and from the ISF into the sweat gland. For instance, if the pressure difference across the sweat gland (ΔP) becomes zero, water flow through the sweat gland ceases, halting sweat secretion. As a result, without the removal of water via the sweat gland, the continued influx of water from the capillaries would increase $P_{\mathrm{ISF}}$ , decreasing the pressure difference $\left(P_{c}-P_{\mathrm{ISF}}\right)$ in Equation 2. This would lead to a decrease in $Q_{\mathrm{water}}$ , the water flow rate from the capillaries into the ISF, until equilibrium is reached when $\left(P_{c}-P_{\mathrm{ISF}}\right)$ becomes zero, and water flow from the blood capillaries stops entirely.

The water flow velocity in the sweat gland compartment, $u_{sg}$ in $m\;s^{-1}$ , is adjusted with a correction factor, $u_{\mathrm{sweat,n}}$ , to account for variations between passive sweating condition and experimental conditions involving sweat stimulation, as shown in Equation (8)

$$u_{sg}=\frac{Q_{\mathrm{water,sg}}\cdot u_{\mathrm{sweat,n}}}{A_{sg}},$$ (8)

where $A_{sg}$ is the luminal area of the sweat gland in $m^{2}$ , $u_{\mathrm{sweat,n}}$ is the normalized experimental sweat velocity, normalized relative to the passive sweat velocity ( $3\times 10^{-4}\;m\;s^{-1}$ (Nie et al., 2018)). This factor represents physiological influences, such as exercise or temperature changes, ensuring the model reflects real-world conditions.

Simultaneously, a fraction of the urea is actively transported into the sweat gland by urea transporters, which move urea against its concentration gradient (Xie et al., 2017). As water enters the sweat gland, a fraction of it is absorbed by the duct wall, reducing the water content and thereby increasing the concentration of urea. Both the passively- and actively-transported urea flow through the sweat gland to the skin surface. This process can be modeled using the diffusion-convection equation as shown in Equation (9)

$$\frac{\partial C_{sg,dil}}{\partial t}=D_{sw}\frac{\partial^{2}C_{sg,dil}}{\partial y_{sg}^{2}}-u_{sg}\frac{\partial C_{sg,dil}}{\partial y_{sg}}+S,$$ (9)

where $C_{sg,dil}$ is the diluted concentration of urea in the sweat gland compartment in $mol\;m^{-3}$ , $D_{sw}$ is the urea diffusion coefficient in sweat in $m^{2}\;s^{-1}$ (Chikode et al., 2021), $y_{sg}$ is the distance between the urea and the entrance of the sweat gland compartment in m , S represents the urea transport rate in $mol\;m^{-3}\;s^{-1}$ , serving as a source term that reflects the active transport of urea mediated by urea transporters. The source term S in this equation is equal in magnitude to the sink term S in Equation 4, representing the same physiological process of the active transport of urea from the ISF compartment to the sweat gland compartment. This ensures mass conservation within the system.

Due to the dilution effect of water entering the sweat gland and the absorption of a fraction of this water by the duct wall (Schwartz et al., 1953), the original concentration of urea in the sweat gland $\left(C_{sg}\right)$ is impacted. This results in a diluted concentration $\left(C_{sg,dil}\right)$ , which can be described as shown in Equation (10)

$$C_{sg,dil}=\frac{C_{sg}}{1+K_{w/u}u_{sg,n}},$$ (10)

Where $K_{w/u}$ is the dimensionless ratio of the volumetric flow rate of water to urea (Kancharla et al., 2019), and $u_{sg,n}$ is the sweat gland water flow velocity $\left(u_{sg}\right)$ normalized to the passive sweat velocity, as defined in Equation 8.

### 2.2 Inverse estimation of blood urea using double-loop optimization

**FIGURE 2:**
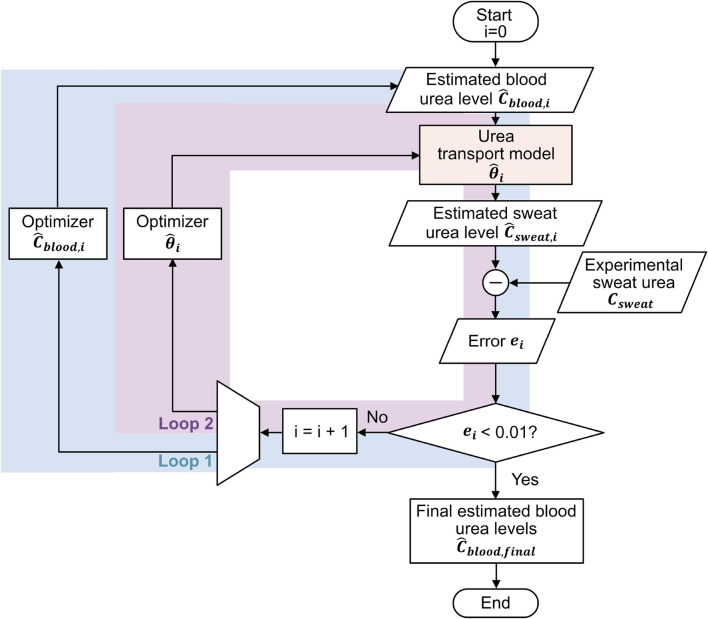
Double-loop optimization flowchart for inverse estimation of blood urea concentrations from sweat urea concentrations: Loop 1 refines the estimated blood urea concentrations, and Loop 2 optimizes the parameter values of the urea transport model, with the two loops alternating sequentially.

As shown in Figure 2, solving the inverse problem by estimating blood urea concentration from sweat urea concentration can be realized using a double-loop optimization strategy, similarly to in our previous work on glucose estimation (Yin et al., 2024). This strategy consists of two intertwined optimization loops: the first loop refines the estimated blood urea concentration $\left({\hat{C}}_{\text{blood},i}\right)$ , while the second loop updates the physiological parameter values $\left(\hat{\theta }\right)$ of the urea transport model. These loops operate iteratively, with one loop succeeding the other to minimize the error between the estimated and experimentally measured sweat urea concentrations.

#### 2.2.1 Initialization of parameters

The optimization process begins with initializing the blood urea concentration $\left({\hat{C}}_{\text{blood},0}\right)$ and the transport model parameters $\left(\hat{\theta }\right)$ . We set ${\hat{C}}_{\text{blood},0}$ to 6.4 $mmol\;L^{-1}$ , which is the average of our experimental post-HD blood urea concentrations. The urea transport model parameters $\hat{\theta }$ , excluding the urea source term (S) , are initially set based on values from literature (see Table 1 for details). The value of S is initialized to 0 due to the lack of reference values in existing studies. Setting S to 0 assumes that urea transport relies solely on passive urea transport mechanisms.

**TABLE 1:** Parameters used in the urea transport model.

Parameter	Unit	Value	Ref
Capillary hydrostatic pressure: $P_{c}$	mmHg	30	Haggerty and Nirmalan (2019)
Capillary hydraulic conductivity: $L_{p,c}$	$m\;s^{-1}\;mmHg^{-1}$	$6.5\times 10^{-10}$	Kellen and Bassingthwaighte (2003)
Dermal clearance constant of urea: $k_{DE}$	$s^{-1}$	$1.2\times 10^{-3}$	Ibrahim et al. (2012)
Diffusion coefficient of urea for gland wall: $D_{sg,wall}$	$m^{2}\;s^{-1}$	$3.01\times 10^{-10}$	Steiner (1981)
Diffusion coefficient of urea in ISF: $D_{\mathrm{ISF}}$	$m^{2}\;s^{-1}$	$9.29\times 10^{-10}$	Steiner (1981)
Diffusion coefficient of cortisol in sweat: $D_{sw}$	$m^{2}\;s^{-1}$	$1.38\times 10^{-9}$	Chikode et al. (2021)
Effective area of sweat gland: $A_{sg}$	$m^{2}$	$1.96\times 10^{-11}$	Hibbs (1958)
Effective surface area of capillary: $A_{c}$	$m^{2}$	$1.5\times 10^{-8}$	Himeno et al. (2016)
Effective cross-sectional area of ISF: $A_{\mathrm{ISF}}$	$m^{2}$	$2.2\times 10^{-8}$	Himeno et al. (2016)
Effective volume of capillary: $V_{p}$	$m^{3}$	$3.02\times 10^{-13}$	Himeno et al. (2016)
Effective volume of ISF: $V_{\mathrm{ISF}}$	$m^{3}$	$6.0\times 10^{-13}$	Himeno et al. (2016)
Interstitial hydrostatic pressure: $P_{\mathrm{ISF}}$	mmHg	−3	Haggerty and Nirmalan (2019)
Inner luminal diameter of sweat gland: d	m	$5\times 10^{-6}$	Hibbs (1958)
Length of sweat gland: L	m	$4\times 10^{-3}$	Wilke et al. (2007)
Ratio of volumetric flow rate of water to urea: $K_{w/u}$	-	2.5	Kancharla et al. (2019)
Thickness of sweat gland wall: $h_{sg}$	m	$5\times 10^{-5}$	Sonner et al. (2015)
Viscosity of water: μ	Pa⋅s	$1\times 10^{-3}$	Kestin et al. (1978)

#### 2.2.2 Optimization processes

Using the initialized parameters, we input ${\hat{C}}_{\text{blood},0}$ into the urea transport model to estimate the sweat urea concentration $\left({\hat{C}}_{\text{sweat},0}\right)$ . The estimated concentration is then compared with the experimentally measured value $\left(C_{\text{sweat}}\right)$ , and the error $\left(e_{i}\right)$ for each i -th iteration is quantified using the squared error as shown in Equation (11)

$$e_{i}={\left(C_{\text{sweat}}-{\hat{C}}_{\text{sweat},i}\right)}^{2},$$ (11)

where $C_{\text{sweat}}$ is the experimentally measured sweat urea concentration, and ${\hat{C}}_{\text{sweat},i}$ is the estimated sweat urea concentration for the same data point in the i -th iteration.

The optimization process continues until $e_{i}<0.01\;{\text{mmol}}^{2}\;{\text{L}}^{-2}$ . At this point, the estimated urea concentration in blood $\left({\hat{C}}_{\text{blood},i}\right)$ is considered the final estimate $\left({\hat{C}}_{\text{blood},final}\right)$ . For a more detailed explanation of the double-loop optimization, please refer to our previous work (Yin et al., 2024).

### 2.3 Data sources and performance metrics

#### 2.3.1 Urea data sources

**FIGURE 3:**
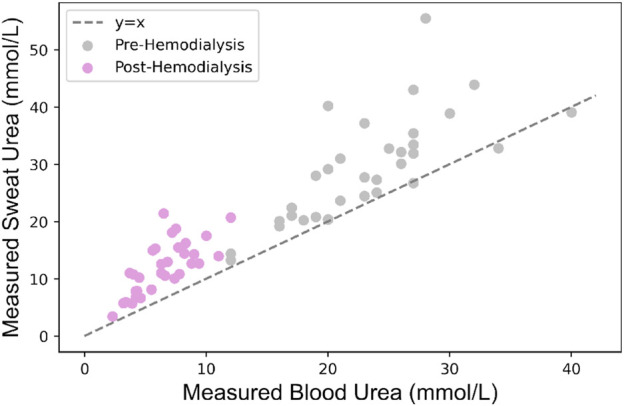
Relationship between measured sweat and blood urea concentrations in patients undergoing HD.

This developed model was validated with data obtained during the observational clinical trial that is registered in the International Clinical Trials Registry Platform (UMIS Study, ID NL9831). The study was approved by the local ethical review board and the medical research Ethics Committees United (MEC-U), and written informed consent was obtained from each patient before participation. This study was carried out in accordance with the principles of the Declaration of Helsinki (Fortaleza, Brazil, October 2013) and in accordance with Dutch law. In the UMIS study, a total of 40 patients were initially included. At the beginning and end of a single HD cycle, both sweat and blood samples were collected from patients. Sweat samples were gathered from the forearm using the Macroduct Advanced Sweat Collection System (Elitechgroup, Logan, Utah, United States), following the manufacturer’s guidelines. Sweat samples were collected under routine HD conditions, where patients typically remained seated or reclined throughout the procedure. While our protocol specified the general sample collection method, we did not explicitly track or control factors such as recent physical activity or dietary intake prior to sweat collection. However, given that participants remained in a resting state during HD and followed standard pre-dialysis dietary guidelines, we did not consider the potential variability in sweat urea concentration due to these factors within this controlled clinical setting. Concurrently, blood samples were obtained while patients were connected to the HD machine, with dialysis flow temporarily reduced from 300 to 100 mL/min. All collected samples were centrifuged within 2 h after collection and urea concentrations ware determined using a kinetic method by the Cobas Pro analyzer (Roche Diagnostics, Rotkreutz, Switzerland). For further information regarding the acquisition protocol, please refer to the study by Adelaars et al. (2024). In this work, 8 patients were excluded due to the absence of at least one required sweat measurement before and/or after HD. The missing values were attributed to the low volume of collected sweat, which hindered the accurate quantification of urea. Consequently, the study proceeded with 32 patient samples, involving paired sweat and blood urea concentrations collected both before and after HD, to validate the model. Figure 3 illustrates the measured sweat urea versus measured blood urea concentrations. Table 2 presents the baseline characteristics of the 32 included patients.

**TABLE 2:** Patient characteristics and measured urea concentrations (N = 32): BMI (body mass index), IQR (interquartile range), GFR (glomerular filtration rate).

Variable	Number	Percentage (%)
Gender, men	23	71.9%
BMI		
< 18	1	3.1%
18–25	10	31.3%
25–30	14	43.8%
> 30	7	21.9%

Variable	Median	IQR (Q1–Q3)
Age, years (min–max)	68 (22–90)	60–77
GFR (mL/min/1.73 m^2^)	7.2	4.8–8.2
Sweat Concentrations (mmol/L)		
Pre-HD	28.57	22.05–33.90
Post-HD	12.01	8.07–15.03
Plasma Concentrations (mmol/L)		
Pre-HD	23.16	19.00–27.00
Post-HD	6.40	4.28–7.90

#### 2.3.2 Performance metrics

The accuracy of our estimation strategy was assessed by computing the Root Mean Square Error (RMSE) and Root Mean Square Percentage Error (RMSPE) (Taraji et al., 2017), and determining the Pearson Correlation Coefficient R , between the estimated and experimental values. To assess the impact of GFR variability on model performance, subjects were stratified into three groups based on their GFR values: low GFR ( < 5 mL/min/1.73 m^2^), mid GFR (5–8 mL/min/1.73 m^2^), and high GFR ( > 8 mL/min/1.73 m^2^). Model performance was evaluated separately for each group using RMSE and RMSPE to determine whether estimation accuracy varied across different levels of residual renal function. Furthermore, a Bland-Altman analysis was conducted to analyze the agreement between the experimental data and estimated results.

To evaluate the significance of the urea source term S in the urea transport model, we compared ${\hat{C}}_{\text{blood},final}$ with that obtained by using a “passive urea transport model”. In the latter, all the model parameters, with exception of the S, were optimized, while S was set at 0 to exclusively account for passive transport mechanisms. Both models were tested using the same experimental datasets to assess the estimation performance. Two-tailed Wilcoxon signed-rank tests were applied to evaluate the statistical significance of the differences in estimation performance between the two models.

#### 2.3.3 Parameter sensitivity analysis for the urea transport model

We conducted a sensitivity analysis to assess the extent to which each parameter of the urea transport model, along with its input $C_{p}$ , can be accurately estimated. To this end, we assessed the sensitivity of the simulated sweat concentration to variations in both model parameters and $C_{p}$ , identifying key variables influencing the model’s output. For most model parameters and the input $C_{p}$ , 100 samples were drawn from a Gaussian distribution centered at each parameter’s literature value, with a standard deviation set to 10% of the mean. The urea source term (S) , whose values are not available from the literature, was set based on the average of our estimates. Matched simulated sweat urea concentrations were computed for each variation, and corresponding coefficients of variation (CV) (Krishnamoorthy and Lee, 2014) were calculated to quantify the model’s sensitivity.

#### 2.3.4 Robustness analysis for the double-loop optimization strategy

To evaluate the robustness of the double-loop optimization strategy to its initial input, the estimated blood urea concentration ${\hat{C}}_{\text{blood},0}$ , its value was varied from 0 to 50 $mmolL^{-1}$ in intervals of 10 $mmolL^{-1}$ . For each variation, the RMSPE of estimation results was computed, and the corresponding CVs were calculated.

## 3 Results

**FIGURE 4:**
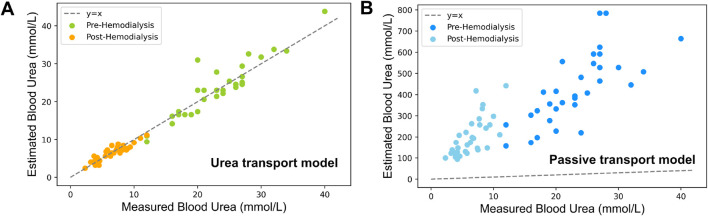
Comparison of estimated vs experimental blood urea concentrations based on measured sweat urea concentrations **(A)** Estimations using the urea transport model incorporating both passive and active transport **(B)** Estimations derived solely from passive transport within the same model.

The plasma urea concentrations estimated using our novel approach are presented in Figure 4A, demonstrating a very high correlation coefficient of 0.98, with a RMSE of 2.9 $mmolL^{-1}$ and a RMSPE of 17.4% acquired before and after HD. In contrast, the results of the passive urea transport model (Figure 4B) showed significantly higher errors with a RMSE and RMSPE of 341.90 $mmolL^{-1}$ and 2,627.17% (p-value < 0.001), while its correlation coefficient was still higher than 0.70, as shown in Table 3. Notably the estimation results of the passive urea transport model, as depicted in Figure 4B, are substantially divergent from the experimental value, indicating a marked overestimation. The model’s estimation performance was further analyzed across different GFR levels. Table 4 summarized the RMSPE and RMSE of blood urea estimation pre- and post-HD. Pre-HD RMSPE ranged from 10.6% to 19.2%, while post-HD RMSPE varied between 13.9% and 19.9%. The absolute difference of RMSPE across GFR groups remained within 9%.

**TABLE 3:** Overview of the urea transport models’ performance.

Model	Transport	Moment	Correlation (95% CI)	RMSE (mmol/L)
Full Urea Transport Model	Active + Passive	Pre HD	0.92 (0.84–0.96)	2.9
		Post HD	0.92 (0.83–0.96)	1.0
		Total	0.98 (0.95–0.99)	2.1
Passive Urea Transport Model	Passive	Pre HD	0.73 (0.51–0.86)	433.4
		Post HD	0.70 (0.46–0.84)	214.4
		Total	0.86 (0.74–0.93)	341.9

**TABLE 4:** Model performance metrics across different GFR groups.

Metric	Low GFR ( < 5)	Mid GFR (5–8)	High GFR ( > 8)
Subject Numbers	9	13	10
Pre-HD RMSPE (%)	19.2	10.6	10.8
Post-HD RMSPE (%)	13.9	19.9	16.9
Pre-HD RMSE (mmol/L)	4.1	2.6	1.7
Post-HD RMSE (mmol/L)	1.0	1.1	0.7

**FIGURE 5:**
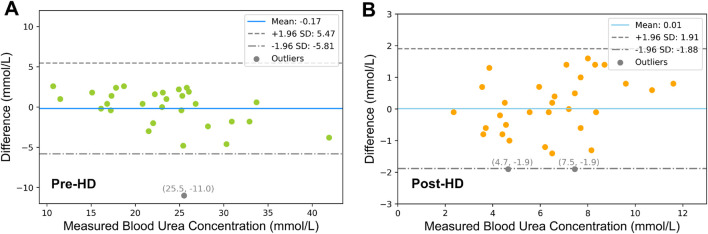
Bland-Altman Analysis of **(A)** Pre-HD and **(B)** Post-HD Estimation Results. The dashed lines represent the 95% limits of agreement, which are derived as the mean difference ± 1.96 times the standard deviation (SD).

The results of the Bland-Altman analysis are shown in Figure 4, assessing the agreement between estimated and experimentally determined blood urea concentrations for both pre-HD (Figure 5A) and post-HD (Figure 5B) data sets. The mean bias was $-0.17\pm 5.64mmolL^{-1}$ (mean ± Limits of Agreement (LoA)) before HD (Pre-HD) and $0.01\pm 1.89mmolL^{-1}$ after HD (Post-HD).

**FIGURE 6:**
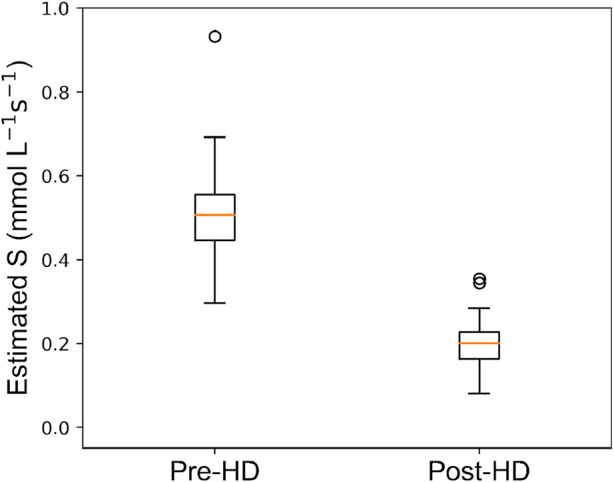
Box plot depicting distribution of estimated urea source (S) values for Pre-HD and Post-HD statuses.

Figure 6 shows the distribution of estimated values for the urea active transport term S pre- and post-HD. For pre-HD, the median (IQR) value of S is 0.51 (0.11) $mmolL^{-1}\;s^{-1}$ . For post-HD, the median (IQR) value of S is of 0.21 (0.06) $mmolL^{-1}\;s^{-1}$ .

Table 5 displays the results of the sensitivity analysis for the parameter values of the urea transport model, reporting only those model parameters that exhibit a CV higher than 1%. Among all parameters, the diffusion coefficient of urea in sweat $D_{sw}$ has the highest CV at 8.8%, followed by the urea source term S with a CV of 7.2%. Additionally, the robustness analysis showed that the CV of the RMSPE for the estimated blood urea concentration $\left({\hat{C}}_{\text{blood},0}\right)$ was 0.06%.

**TABLE 5:** Sensitivity analysis of key parameters in the urea transport model.

Parameters	Symbol	Unit	Coefficient of variation (CV)
Diffusion coefficient of urea in sweat	$D_{\text{sw}}$	${\text{m}}^{2}{\text{s}}^{-1}$	8.8%
Urea source term	S	$\text{mol}{\text{m}}^{-3}{\text{s}}^{-1}$	7.2%
Ratio of volumetric flow rate of water to urea	$K_{\text{w/u}}$	-	4.7%
Urea concentration in plasma	$C_{\text{p}}$	$\text{mol}{\text{m}}^{-3}$	2.1%
Thickness of sweat gland wall	$h_{\text{sg}}$	m	1.1%

## 4 Discussion

In this study, we introduced a novel urea transport model to describe the process of urea transport from blood to sweat through a single sweat gland, considering both passive and active transport mechanisms.

### 4.1 Model accuracy and robustness

The urea concentration before and after HD was accurately estimated by the proposed urea transport model, with an average RMSE of 2.1 $mmolL^{-1}$ and a correlation coefficient of 0.98 (95% CI: 0.95–0.99). Across all observed GFR levels, the model exhibited consistent estimation performance, with RMSPE variability remaining below 9% across different GFR groups. This suggests that the relationship between sweat and blood urea concentrations remains robust within all observed GFR levels, indicating that residual renal function does not significantly impact the validity of the proposed model. Additionally, since HD sessions occurred at different times of the day, any existing circadian variation was inherently reflected in the measured blood urea values. This suggests that our model captures urea transport dynamics across varying time points without requiring explicit circadian adjustments. Moreover, in dialysis patients, the impact of treatment schedules on urea levels may partially overshadow circadian fluctuations, further supporting the robustness of our approach in real-world clinical applications. Several physiological and environmental factors influence sweat secretion, including variations in sweat gland density across anatomical sites, age-related changes in gland function, and external conditions such as temperature and humidity (Baker, 2019; Schmidt et al., 2022; Taylor and Machado-Moreira, 2013). These factors primarily affect sweat rate rather than the underlying urea transport mechanisms. Since our model explicitly incorporates each individual’s measured sweat rate as an input parameter, it inherently accounts for these variations, ensuring robust estimation of blood urea concentrations across different conditions.

Further validation of our urea transport model was performed using Bland-Altman analysis to compare the model estimates with experimental plasma values. The analysis revealed a clinically irrelevant bias of −0.17 $mmolL^{-1}$ for pre-HD and −0.01 $mmolL^{-1}$ for post-HD that are well within the analytical inaccuracies of urea assays and biological variation (“EFLM Biological Variation,” n. d.). These near-zero values underscore the high accuracy of our proposed urea transport model.

Beyond evaluating the accuracy and performance of our model, we also investigated its robustness under varying initial conditions to ensure reliability across a broad spectrum of clinical scenarios. Specifically, the initial value of the model input, i.e., the estimated blood urea concentration $\left({\hat{C}}_{\text{blood},0}\right)$ , was varied from 0 to 50 $mmolL^{-1}$ . The resulting coefficient of variation was 0.06%, indicating that changes in input initialization had only a minimal effect on the final estimation of blood urea concentration. This finding demonstrates the negligible impact of input variability on the model outcomes, underscoring its robustness against local error minima. Such stability not only enhances the reproducibility of the model predictions but also ensures reliable estimations across diverse clinical contexts.

### 4.2 Mechanistic insights into urea transport

The strategy proposed in this paper introduces an active transport mechanism of urea through the model parameter S . The presence of active transporters is supported by existing literature. Consistent with previous research from our group (Adelaars et al., 2024) and others (Bulmer, 1957; Al-Tamer et al., 1997; Adelaars et al., 2024), we found that experimental urea concentrations in sweat are higher than those in plasma. There is only very limited literature about the possible urea transport mechanisms that could explain the elevation in sweat. Although several dated studies hypothesize an epidermal source of urea (Brusilow, 1967), these were not scientifically confirmed. More recently, Xie et al. (2017) identified increased expression of active urea transporters (UTs) in the membranes of sweat glands in uremic patients. This suggests an enhanced active transport of urea into sweat under uremic conditions. Furthermore, existing literature confirms the presence of aquaporins-5 (AQP-5) in the sweat gland (Nejsum et al., 2002; Inoue et al., 2013), indicating the possibility of either active dilution or desiccation of urea within the sweat gland through active water transport. This observation also aligns with our introduction of the parameter S in the model. The introduction of the source term S in our urea transport model offers insights into the dynamics of urea transport, particularly in response to the physiological changes induced by HD. Pre-HD, when blood urea concentrations are elevated, the mean value of S was also higher, likely due to increased interactions between urea molecules and their transporters. Conversely, post-HD analysis revealed significantly lower S values, consistent with the expected reduction in urea transport activity following dialysis.

The critical role of S in our urea transport model is further clarified through the presented sensitivity analysis (Table 5). With a CV of 7.2%, S is the second most influential parameter affecting the performance of our proposed urea transport model. The diffusion coefficient of urea in sweat $\left(D_{sw}\right)$ emerged as the most sensitive parameter, with a CV of 8.8%. These findings highlight the model’s sensitivity to changes in different physiological conditions, as reflected by variations in key parameters. The diffusion coefficient $D_{sw}$ represents the ease by which urea molecules move in the sweat gland. Clinically, this parameter may vary due to differences in glandular function, which are known to change with age (Schmidt et al., 2022). Lower $D_{sw}$ values might suggest reduced gland function, commonly observed in aging individuals. Similarly, the urea source term S represents the active transport of urea into the sweat gland, influenced by transporter expression. Elevated S values may reflect upregulated transporter activity, potentially associated with uremic conditions, as suggested by Xie et al. (2017). These findings emphasize the importance of personalized parameter estimation to ensure reliable model performance.

### 4.3 Comparison with existing models

When considering a model accounting for passive urea transport only, the results show a significant under performance due to a systematic overestimation of blood urea concentrations. Such an overestimation by the passive urea transport model is attributed to its exclusive reliance on the passive transport mechanisms of diffusion and convection. When these are the sole mechanisms considered, the influx of water into the sweat glands leads to a dilution effect that notably reduces the urea concentrations in sweat, which is responsible for the observed overestimation. Our urea transport model addresses this limitation by incorporating an additional term, S , which represents the active transport of urea mediated by urea transporters. This adjustment effectively corrects for the dilution effect and its associated overestimation.

The only existing research focused on estimating plasma urea from sweat urea concentrations was performed by our group using linear regression analysis (Adelaars et al., 2024). Adelaars et al. (2024) indicated an average RMSE and RMSPE of 4.3 $mmolL^{-1}$ and 37.7%, respectively, which are doubled with respect to the 2.1 $mmolL^{-1}$ and 15.6% achieved by our work. Adelaars et al. (2024) also reported a Spearman’s correlation coefficient of 0.92 (95% CI: 0.88–0.95). In comparison, our model exhibited superior performance, achieving a Spearman’s correlation coefficient of 0.98 (95% CI: 0.95–0.99). These findings indicate that the urea transport model proposed in this study more accurately estimates blood urea concentrations from sweat measurements.

### 4.4 Clinical implications and future directions

Our study demonstrates the efficacy of kinetic modeling in elucidating the dynamics of urea across body fluid and its relationship with plasma concentrations both during and between HD sessions. This methodology could enable more personalized monitoring and management of renal failure patients who are not yet undergoing HD treatment, potentially postponing the clinical need for HD. It also holds promise for extending its application to predict plasma concentrations of other clinically relevant biomarkers using sweat-sensing technology.

Our urea transport model represents an important step towards clinical implementation of sweat-based urea monitoring, which has been hampered by the unclear relationship between urea concentrations in blood and sweat. This advancement could broaden the scope of non-invasive patient monitoring, paving the way for innovative, patient-friendly tools that enhance clinical practice beyond ESRD. Moreover, our proposed method not only estimates blood urea concentrations, but also provides physiologically meaningful model parameters, offering insights for improved diagnosis and decision-making, and contributing to achieving more personalized and effective patient care. Clinically, this modeling framework could be integrated into sweat-sensing devices for home-based urea monitoring, reducing the need for frequent blood draws and enabling more frequent assessments of dialysis adequacy. In home dialysis settings, such non-invasive technology could improve patient comfort and adherence, allowing earlier detection of inadequate dialysis and timely treatment adjustments. This approach may ultimately enhance quality of life for ESRD patients by minimizing hospital visits and personalizing therapy schedules.

With this model, we have taken the initial steps towards enabling the clinical interpretation of sweat urea concentrations, validated with data from 32 patients undergoing HD. However, the study presents some limitations. First, the computational demand of our approach is relatively high. Multiple simulation iterations lead to computation times of approximately 5–10 min per data point, which may hinder its application for real-time monitoring in clinical settings. Future work should focus on developing more efficient algorithms or streamlined simulation techniques to facilitate real-time estimation. Second, the dataset used in this study is relatively small and lacks healthy subjects and patients in the early stages of kidney disease, potentially limiting the generalizability of the model beyond individuals with ESRD. Although the dataset includes diverse demographic and physiological factors, such as sex, age (22–90 years), and BMI (18–30), it does not explicitly categorize factors like concomitant drug use and ethnicity, whose influence on sweat physiology remains unexamined. Findings by Xie et al. (Xie et al., 2017) suggest a decrease in AQP5 expression in uremic patients compared to healthy individuals, indicating that transport mechanisms for urea and water may vary across populations. Moreover, the study was conducted in a relatively controlled clinical setting (HD) and did not systematically assess the impact of demographic and environmental factors, such as temperature and humidity, on model performance. Future studies should validate the model in a broader population, including both healthy individuals and patients at earlier stages of kidney disease, while investigating how demographic and environmental variables may affect model parameters. Additionally, 8 patients were excluded due to insufficient sweat volume for accurate urea quantification. While this represented only 20% of the study cohort, it underscores a potential limitation in real-world applications, particularly for individuals with limited sweat production. However, current research is focusing on the development of microfluidic-based devices for collection of low sweat volumes which should be better investigated in the future to ensure reliable measurements of sweat under various conditions (Moonen et al., 2024). Finally, while this proposed strategy offers promise for non-invasive urea monitoring, its adoption into practice may face certain obstacles. Home-based monitoring devices, while enabling convenient and frequent assessments, can be costly and may be unaffordable for some patients. Besides, integrating these systems into clinical workflows may require staff training and process adjustments. Overcoming these challenges will require affordable device design, simplified workflows, and user-friendly implementation for effective integration into routine care.

Although measuring urea concentrations in sweat has become feasible, research towards the clinical significance of sweat urea remains limited. This study introduces a novel urea transport model that includes both active and passive transport of urea from blood to sweat. By accurately estimating blood urea concentrations from sweat measurements, our model bridges a crucial gap, enabling the translation of sweat urea data into clinical insights. This advancement facilitates the progression toward remote, non-invasive urea monitoring in patients with ESRD using sweat-sensing technology.

## Data Availability

The original contributions presented in the study are included in the article/supplementary material, further inquiries can be directed to the corresponding author.
